# Recent Studies on the Speciation and Determination of Mercury in Different Environmental Matrices Using Various Analytical Techniques

**DOI:** 10.1155/2017/3624015

**Published:** 2017-11-20

**Authors:** Lakshmi Narayana Suvarapu, Sung-Ok Baek

**Affiliations:** Department of Environmental Engineering, Yeungnam University, Gyeongsan-Si 38541, Republic of Korea

## Abstract

This paper reviews the current research on the speciation and determination of mercury by various analytical techniques, including the atomic absorption spectrometry (AAS), voltammetry, inductively coupled plasma optical emission spectrometry (ICP-OES), ICP-mass spectrometry (MS), atomic fluorescence spectrometry (AFS), spectrophotometry, spectrofluorometry, and high performance liquid chromatography (HPLC). Approximately 96 research papers on the speciation and determination of mercury by various analytical instruments published in international journals since 2015 were reviewed. All analytical parameters, including the limits of detection, linearity range, quality assurance and control, applicability, and interfering ions, evaluated in the reviewed articles were tabulated. In this review, we found a lack of information in speciation studies of mercury in recent years. Another important conclusion from this review was that there were few studies regarding the concentration of mercury in the atmosphere.

## 1. Introduction

Mercury is the only metal that exists in a liquid state among the elements in our modern periodic table. Determination and speciation studies of mercury attract researchers because of the toxicity of mercury to humans, as well as to other animals in the food web. The difference between the toxicity of mercury and that of other metals is that mercury easily accumulates in organisms. A few studies have reported bioaccumulation of mercury in various aquatic animals, such as fishes, pelagic seabirds, and earthworms [1–9].

This section describes the sources and fate of mercury in the environment and its toxicity.

### 1.1. Sources and Fate of Mercury in the Environment

Mercury can enter the environment from natural and/or anthropogenic sources. Natural sources of mercury include volcanoes, forest fires, cinnabar (ore), and fossil fuels, such as coal and petroleum. Anthropogenic sources are numerous; a large number of human activities are responsible for mercury deposition in the environment. Anthropogenic sources of mercury are landfills, dental preparations, and combustion processes, such as coal-fired power generation, medicinal waste incinerators, and municipal waste combustion. Manufacture of metals, alkali, and cement also releases mercury into the environment [10]. Anthropogenic sources are related to human activities in contaminated locations. This section describes the sources of mercury in the environment, reported from various parts of the world. Zhuang and Gao [11] reported higher concentrations of mercury in riverine sediments than in marine sediments and concluded that river transportation was the main source of mercury in southwestern Laizhou Bay, China. Kwon et al. [12] found that watershed runoff was the primary route of mercury transfer between lakes and forests.

Xu et al. [13] revealed that mercury concentration in soil has recently increased 3–10 times because of the combustion of fossil fuels combined with long-range atmospheric transportation processes. Shamsipur et al. [14] and Rajabi et al. [15] reported the determination of mercury in water samples using spectrometric and electrochemical techniques, respectively. Han et al. [16] found lower concentrations of wet-deposited mercury in forest areas of South Korea during summer because of precipitation. The concentration of mercury in the atmosphere was influenced by the seasons. In the atmosphere, coal combustion was the major source of gaseous elemental mercury, but traffic emissions contributed particulate mercury. Domestic pollutants are major sources of reactive gaseous mercury [17].

### 1.2. Toxicity and Health Implications of Mercury and Its Different Species

Researchers determine the concentration of mercury in environmental segments because of its toxic nature. Numerous journal articles have been published regarding the toxicity of mercury and its different forms. Yoshida et al. [18] reported on its neurobehavioral toxicity in mice exposed to low-level mercury vapor and methylmercury. Bucio et al. [19] studied the toxicity of mercury in a human hepatic cell line (WRL-68 cells). Results of this study indicated that higher doses of mercury cause cytotoxic effects with the release of lactate dehydrogenase from cells. Mercury exposure can cause neurodegeneration with oxidative stress in mitochondria [20]. Occupational exposure to mercury in workers of a fluorescent lamp factory in Egypt resulted in symptoms including emotional ability, memory changes, neuromuscular changes, and performance deficits in tests of cognitive function [21]. Mercury(II) and methylmercury toxicity can inhibit the human thioredoxin system. Mercury inhibition is selective for the thioredoxin system; mercury binds with selenol-thiol in the active sites of thioredoxin reductase [22]. Methylmercury reacts with the sulfhydryl groups throughout the human body and influences the functions of cellular and subcellular structures. Mercury toxicity in various forms can cause thyroid dysfunction because of the inhibition of 5′deiodonases, spermatogenesis because of accumulation in the testicles, and atrophy and capillary damage in thigh muscles [23]. Tonazzi et al. found a correlation between mitochondrial carnitine-acylcarnitine transporter inactivation and mercury toxicity in animals [24]. Mercury toxicity in humans can cause numerous neurological or psychiatric disorders not limited to autism spectrum disorders, Alzheimer's disease, Parkinson's disease, epilepsy, depression, and tremor. In rats, mercury(II) toxicity affects the central neurons and leads to cytoskeleton instability [25]. Exposure to organic forms of mercury, such as ethylmercury or methylmercury, can cause neurotoxic effects in developing mammals. Ethylmercury exposure in humans occurs because of immunization with thimerosal-containing vaccines [26]. The toxicity of mercury not only is limited to neurological effects in humans, but also causes vascular effects, such as increased oxidative stress and inflammation, thrombosis, endothelial dysfunction, dyslipidemia, and immune and mitochondrial dysfunctions [27]. Overall, the toxicity of mercury in animals and humans affects the cardiovascular, hematological, pulmonary, renal, immunological, neurological, endocrine, reproductive, and embryonic systems [28].

Plants are exposed to mercury compounds through the administration of antifungal agents. The toxicity of mercury affects seed germination, growth, and development in higher plants. It also causes the breakdown of photosynthesis by affecting chlorophyll and magnesium molecules [29]. Mercury toxicity induces oxidative stress in growing cucumber seedlings and results in plant injury [30]. Mercury that has accumulated in different forms within plants can cause phytotoxicity and impair numerous metabolic processes, including nutrient uptake, water status, and photosynthesis [31].

In this present study, we reviewed speciation and determination studies of mercury in different environmental samples using various analytical techniques, including the atomic absorption spectrometry (AAS), voltammetry, inductively coupled plasma optical emission spectrometry (ICP-OES), ICP-mass spectrometry (MS), atomic fluorescence spectrometry (AFS), spectrophotometry, spectrofluorometry, and high performance liquid chromatography (HPLC). Over 96 research papers published since 2015 in reputable international journals were reviewed. This review clearly summarizes the current research on speciation and determination studies of mercury from locations worldwide.

## 2. Reviews of the Determination of Mercury

The toxic nature of mercury and its different species encourage researchers to determine their concentrations in different environmental samples. Recently, a number of reviews were published concerning the determination of mercury, which described various factors regarding the concentrations of mercury in the environment. This section summarizes recent reviews of the determination of mercury.

Hanna et al. [32] reviewed the concentrations of mercury in freshwater fishes of Africa. They reviewed 30 identified studies in which the authors collected 407 Hg concentrations from 166 fish species, 10 types of invertebrates, and various plankton species from 12 countries in Africa. The authors concluded there was a lack of data regarding Hg concentrations in African countries. However, based on available data, Hg concentrations were lower than that of the World Health Organization (WHO) recommendations for commercially available fishes in Africa. Ferreira et al. [33] reviewed analytical strategies of sample preparation for the determination of mercury in food samples using a cold vapor atomic absorption spectrometry (CV-AAS), cold vapor atomic fluorescence spectrometry (CV-AFS), inductively couple plasma mass spectrometry (ICP-MS), voltammetry, and neutron activation analysis. Based on the reviewed papers, they concluded that the determination of mercury and its species in food samples with CV-AFS or CV-AAS was simpler and less expensive than other methods.

Colorimetric and visual assay determination of Hg(II) based on gold nanoparticles, fluorescent gold nanoparticles, gold nanorods, gold nanoflowers, and gold nanostars was reviewed by Chansuvarn et al. [34]. They reported that gold nanoparticles were the most promising luminescent nanomaterials for the detection of Hg(II) because of high selectivity and ultrasensitivity. Regarding analytical instruments, the UV-visible spectrophotometer was cost-effective for limited-budget laboratories for real-time monitoring of Hg(II) in environmental samples. Ariya et al. [35] reviewed physiochemical and biogeochemical transformation of mercury in the atmosphere and at atmospheric interfaces. The authors described the analytical methodology for speciation of mercury in the atmosphere, exchange of Hg between the atmosphere and aquatic interfaces, and exchange of Hg between the atmosphere and terrestrial environments. Shrivastava et al. [36] reviewed Hg detoxification mechanisms in plants. The authors found that Hg had harmful toxic effects on the molecular and physiobiochemical behavior of plants. Another important conclusion of this study was that most research was conducted on seed germination and shoot, root, and leaf morphology. Duarte et al. [37] reviewed the utility of disposable sensors for the detection of lead(II), cadmium(II), and mercury(II) in the environment. The paper describes analytical performance and the effect of certain factors, such as immobilization procedures and surface modification, on the analytical characteristics of the sensors. The authors found that disposable sensors used for single measurements of lead(II), cadmium(II), and mercury (II) in environmental samples had adequate intersensor reproducibility, sensitivity, and selectivity and very low detection limits. They concluded that the modified carbon paste electrode provided better determination of Hg(II) and As(III), because of superior deposition with linear and improved responses under the set of studied conditions. The authors stated that the disadvantages of using macroelectrodes included their expense and that they suffered from surface fouling even though they provided better sensitivity and selectivity for the determination of Hg(II) and As(III).

Jagtap and Maher [38] reviewed the measurement of mercury species in sediments and soils by HPLC coupled with ICP-MS. The authors recommended the extraction of Hg species for determination by distillation or use of 2-mercaptoethanol. They also recommended usage of C8 as the stationary phase and 2-mercaptoethanol as the mobile phase in HPLC for accurate quantification of methyl mercury in presence of large amounts of Hg(II). Gustin et al. [39] reviewed the measurement and modeling of mercury in the atmosphere. These authors reported that mercury in the atmosphere can exist in three different forms, gaseous elemental mercury (GEM), gaseous oxidized mercury (GOM), and particulate bound mercury (PBM). Among these forms, there was relative confidence in GEM measurements only, whereas the remaining two forms were less understood. These authors concluded that only through the comparison of multiple calibrated measurements could the results be determined accurately. McLagan et al. [40] reviewed passive air sampling of GEM in the atmosphere. They found that the performance of the passive air sampling method must be validated against active air monitoring systems with satisfactory precision and accuracy. Jackson and Punshon [41] reviewed recent advances in the measurements of arsenic, cadmium, and mercury in rice and other food materials. They described the challenges, state-of-the-art methods, and usage of spatially resolved techniques for arsenic and mercury within rice grains. However, this review focused mainly on the determination and speciation studies of arsenic rather than mercury. Duan and Zhan [42] reviewed recent use of nanomaterials-based (noble metal nanoparticles, fluorescent metal nanoclusters, semiconductors quantum dots, and carbon nanodots) optical sensors for Hg(II) detection. They concluded that the advantages of using nanomaterials for Hg(II) detection and removal included higher sensitivity and selectivity, simpler and more rapid procedures, and lower cost than that of conventional methods. Sun et al. [43] reviewed the recent progress in detection of Hg using surface enhanced Raman spectroscopy (SERS). They stated that substantial enhancement in detectable Raman signals coupled with a unique nanoparticle-based approach made SERS a powerful tool for the detection of Hg(II). Suvarapu and Baek [44] reviewed the speciation and determination of mercury using various analytical techniques. They discussed research papers published during 2013-2014 on these topics.

## 3. Discussion

In recent years, a large number of research articles were published regarding the determination and speciation of mercury using various analytical techniques. We have divided this section into four parts based on the analytical techniques used in the determination of type and levels of mercury. They are (i) spectrometric techniques (AAS, AFS, ICP-OES, MS, spectrophotometry, and spectrofluorometry), (ii) electrochemical techniques (voltammetry and potentiometry), and (iii) miscellaneous techniques.

The determination and speciation of mercury using spectrometric techniques, such as AAS, AFS, ICP-OES, ICP-MS, spectrophotometry, and spectrofluorometry are presented in Table 1.  Table 2 represents the determination of mercury using electrochemical techniques, and Table 3 represents the determination of mercury using miscellaneous techniques. In these tables, we have incorporated all the analytical variables of merit, such as limits of detection, linearity range, quality control and assurance, applicability (analyzed samples), and interference reported in the determination of mercury.

**Figure 1:**
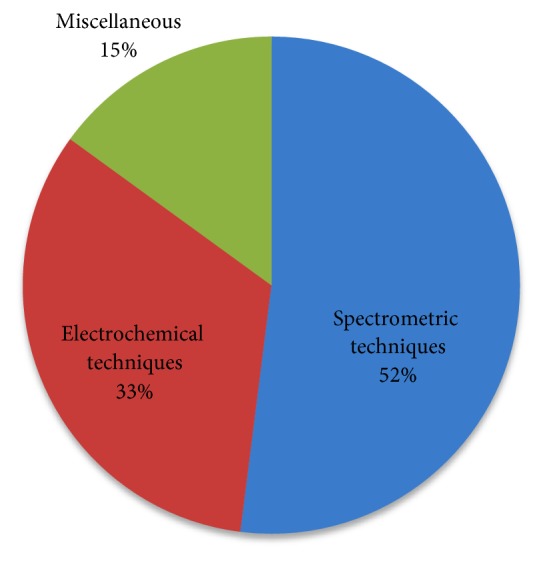
Determination and speciation of mercury using various analytical techniques.

Regarding the usage of analytical techniques in the determination studies of mercury, as presented in Figure 1, 52.00% used spectrometric techniques, such as AAS, AFS, ICP-OES, ICP-MS, spectrometry, and spectrofluorometry, 30.00% used electrochemical techniques, such as a voltammetry and potentiometry, and 15.00% used miscellaneous techniques.

The analytical variables of merit, such as limits of detection and linearity, quality control and assurance studies, applicability to natural samples, and interference, are indicative of the validity of the method. Detection limit indicates the lowest level of analyte that can be detected using the method. A few studies [45–51] reported lowest levels down to picograms of mercury. Methods used in these studies can be considered highly sensitive because of their low detection limits. The lower detection limits were primarily obtained with ICP-MS and CV-AFS. On the other hand, spectrophotometers and spectrofluorometers can provide reasonable sensitivity, and they are inexpensive compared to ICP instruments. Linearity describes the range within which the method can determine analyte concentrations. Most of the electrochemical methods and spectrophotometry and spectrofluorometry methods determined the linearity range of analyte concentrations.

Two very important analytical parameters, in the determination of mercury, are quality assurance (QA) and quality control (QC). The validity and reliability of the data produced by the researchers depend on the quantification of these variables. Quality assurance studies can be performed by testing the accuracy of the data obtained against standard reference materials (SRMs) provided by the National Institute of Standards and Technology (NIST, USA) or certified reference materials (CRMs) provided by various reputable institutes or organizations. Quality control can be determined by measuring the precision of the data (repeatability and sensitivity) obtained by each method. The precision of the data can be obtained in many ways, such as the analysis of replicates, interlab comparison of data, and relative standard deviation (RSD) of blank or standard material analysis [52]. Regarding QA, in the reviewed papers, a few [45, 53–57] reported the analysis of SRMs to compare with the results of their methods. The results obtained with the measuring of SRMs give validity to the obtained data. The other alternative to measure the accuracy of the data is the analysis of CRMs. A large number of studies [45, 47, 55, 56, 58–67] reported the analysis of CRMs to validate their data. Regarding QC, most of the studies reported the RSD values for replicate sample analysis and/or standard materials analysis. Overall, most researchers were aware of the quality of their data, whereas a few [46, 51, 68–87] did not report any QA or QC values, which negatively affected the reliability of their data.

The validity of analytical methods can be enhanced by applicability to natural samples. Regarding the analysis of natural samples, most of the reviewed papers analyzed water samples, such as those from rivers, lakes, seas, groundwater, and spiked water and wastewater. Following water samples, the most frequently analyzed material for mercury was seafood samples, such as fish, shrimp, and seaweed. A few studies reported the determination of mercury in various environmental samples, such as petroleum hydrocarbons [88], human hair [89, 90], phosphate fertilizers [53], glycerin [91], sediments [55, 92, 93], cosmetics [94], living cells [66, 95], and tobacco [67]. However, a few methods [74, 81, 87, 96–101] did not report their applicability to natural samples. We found that very few authors [46] determined mercury content in the atmosphere. Because of the difficulty in sampling and analysis, most authors did not address this issue.

Another important aspect of analytical parameters of the methods is interference. Interference of other ions in the determination of mercury levels is very important, particularly when those methods are applied to the analysis of natural samples. Natural samples are typically a complex of different ions; the selectivity of the method is very important in the determination of mercury in environmental samples. In this respect, electrochemical methods had a higher degree of selectivity and did not suffer from interference from other ions. Determination of the level of interference was not performed by a large number of authors [45–47, 53, 55, 59, 60, 62, 63, 69, 88, 91, 92, 102–106] who determined mercury with spectrometric instruments. However, those using electrochemical instruments, spectrophotometers, or spectrofluorometers largely reported the level of interfering ion(s).

Toxicity of mercury depends upon its chemical form. For example, methylmercury is more toxic than inorganic mercury. Speciation studies revealed the exact toxicity of mercury in environmental samples. However, very few authors [88, 90, 92, 107] reported the speciation of mercury, and most authors determined the level of inorganic mercury. More than 90% of studies using electrochemical methods or spectrophotometry and spectrofluorometry techniques determined divalent inorganic mercury and did not report speciation. However, a few reported [51, 53, 55, 57, 58, 60–62, 66–68, 85, 86, 93, 102–106, 108, 109] total mercury content in various samples, which does not accurately predict toxicity based on its concentration.

## 4. Conclusions

The present study reviewed research articles published in recent years (2015-2016) involving determination and speciation of mercury using various analytical instruments. Approximately 100 research papers were reviewed and all the analytical parameters established in their studies were tabulated. Our study concluded that most of researchers used spectrometric instruments for the determination of mercury in different environmental samples. We addressed the quality of the data based on reported QA and QC data by the authors. Another important finding from this review was that most researchers measured inorganic mercury or total mercury, whereas only a few reported speciation of mercury. Speciation studies are very important in the accurate prediction of the toxicity of the mercury in the environment because mercury toxicity depends on its chemical form. Because of the difficulty in sampling and analysis, most researchers did not report the concentrations of mercury in the atmosphere. We conclude by stating that speciation studies and the determination of mercury in the atmosphere should receive greater attention in the future.

## Figures and Tables

**Table 1:** Analytical parameters of reviewed research papers involving speciation and determination of mercury by spectrometric instruments (AAS, ICP-OES, ICP-MS, AFS, spectrophotometer, and spectrofluorometer).

Analyte	Method	Supporting media	Analytical instrument	LOD	Linearity range	QA/QC studies	Analyzed samples	Interference study	Ref
Hg(II)	Fluorescence	Dithioacetal-substituted triphenylimidazole	Fluorescence spectrometer	4.3 nM	0–10 *μ*M	Sensitivity and selectivity of the method evaluated	—	Cations, such as Ag^+^, CO^2+^, K^+^, Sn^2+^, Cu^2+^, Ni^2+^, Mn^2+^, Na^+^, Ca^2+^, Mg^2+^, Pb^2+^, Fe^3+^, and Cd^2+^ did not interfere in determination of Hg^2+^	[96]

Hg(II)	Fluorescence	2-Aminoethyl piperazine and 4-chloro-7-nitrobenz-2-oxa-1,3-diazole	Fluorescence spectrometer	0.57 *μ*M	0.00–16.6 *μ*M	The method is selective over 18 metal ions. Recoveries of Hg(II) in water samples in the range of 95–98.2%	Water samples	Either no or a small fluorescence signal was observed for Na^2+^, K^2+^, Mg^2+^, Ca^2+^, Al^3+^, Ga^3+^, In^3+^, Cr^3+^, Mn^2+^, Fe^2+^, Fe^3+^, Co^2+^, Ni^2+^, Cu^2+^, Zn^2+^ Cd^2+^, and Pb^2+^ ions	[110]

GEM	Acid digestion	Teflon filters	ICP-MS	0.015 M	—	SRM 1633c was analyzed. Recoveries were in the range of 84–112%	Particulate matter	—	[45]

Hg(II)	CVG	LiAlH_4_, SnCl_2_/THB	HG-AFS	0.0004 *μ*M		The RSD values were less than 7.0% for 11 measurements. CRMs were analyzed	Soil, water, and human hair samples	10 mg L^−1^ for Fe^3+^, 20 mg L^−1^ for Co^2+^, 10 mg L^−1^ for Ni^2+^, and 20 mg L^−1^ for Cu2+. For arsenic and antimony, no interference from 25 mg L^−1^ Cu^2+^, Co^2+^, or Ni^2+^ was observed	[89]

Hg(II)	Photochemical vapor generation	—	ICP-OES	0.006 *μ*M	Up to 0.25 *μ*M	Recoveries of Hg(II) in reals samples were in the range of 79–112%	Petroleum production water	K^+^, Ba^2+^, Sr^2+^, Mg^2+^, Cu^2+^, Pb^2+^, and Zn^2+^ were over the concentration ranges studied, whereas the presence of Ca^2+^, Ni^2+^, Sb^3+^, As^3+^, Se^4+^, Fe^3+^, and Cr^3+^ was significant	[111]

Hg(II)	Acid digestion	—	ICP-MS	—	0.0005–0.5 *μ*M	Precision of the method for Hg2+ is 5.6% with six replicates	Fish samples	Hg(II) was analyzed along with Cd^2+^ and Pb^2+^	[112]

Total Hg	Wet digestion	—	AAS	—	—	—	Fish samples	Hg(II) was analyzed along with Cd^2+^ and Pb^2+^	[68]

Hg speciation	Acid digestion	—	ICP-MS	0.00004 *μ*l/L	—	NIST 612 was analyzed	Petroleum hydrocarbons	—	[88]

Total Hg	Chemical vapor generation	Nonionic surfactants	AFS	22.5 *μ*M	—	CRMs (GBW08603, GBW(E) 080401 and GBW(E)080402) were analyzed	Natural water samples	Severe interference of cations was observed at 10 mg L^−1^ concentration	[58]

Total Hg	Sequential extraction	—	CV-AAS	—	—	Comparison of the results with other methods was performed	Thar coal	—	[102]

Hg(II)	Aptasensor	Core-shell Ag@SiO2 nanoparticles	AFS	0.33 nM	0–1.2, 1.2–14 nM	Recoveries were over 94% for the determination of Hg(II) in real samples. The RSD values for Hg(II) determinations were lower than 5.1%	Real water samples	Selective in presence of Cd^2+^, Pb^2+^, Zn^2+^, Mn^2+^, Co^2+^, Fe^2+^, Cu^2+^, and Ag^+^	[113]

Hg(II)	Chemosensor	Porphyrin-thymine conjugates	Luminescence spectrometer	6.7 nM	—	Method reversibility was reported	—	Interference of Zn^2^+, Cu^2+^, Ni^2+^, Mn^2+^, Mg^2+^, Pb^2+^, and Cd^2+^ was inhibited	[97]

Hg	PVG and CVG	NaBH_4_/SnCl_2_	PVG-AAS, CV-AAS (NaBH_4_) and CV-AAS (SnCl_2_)	0.0006, 0.0005, and 0.0002 *μ*M	0.012–0.05 *μ*M	The accuracy was evaluated by assessing recoveries in spiked samples which were in the range of 84–108.3%	Glycerin samples	—	[91]

Total Hg	Solid sampling analysis	—	GF-AAS	0.0014 *μ*l/L	—	SRM (NIST-695) was analyzed. The RSD values were better than 8.2% for five replicates	Phosphate fertilizers	—	[53]

DGM, TGM	—	—	CV-AFS	1.35 × 10^−8^ *μ*M (DGM), 1.35 × 10^−8^ *μ*M (TGM)	—		Air-sea interface of Minamata	—	[46]

Methyl Hg	Distillation and solvent extraction	KBr/CuSO_4_	GC-ICP-MS	2.0 × 10^−5 ^*μ*g g^−1^	—	CRM of ERM-CC580 was analyzed	Peat soil	—	[59]

Total Hg	Ultrasound extraction	NaBH_4_/isoamyl alcohol, thiourea	CV-AAS	70 *μ*M	—	The RSD values of Hg determinations in vinegar was less than 8.11%	Vinegar	Vanadium also determined in the same samples	[108]

Total Hg	Slurry sampling		CV-AAS	150 *μ*M	—	The RSD values in the mercury determinations was less than 10.89%. The recoveries were in the range of 85–106%	Iron supplement	—	[103]

Total Hg		NaBH_4_	AES	0.00004 *μ*g g^−1^	—	—	Sea food	—	[69]

Hg(II)	Preconcentration	Metal-organic frame work	CV-AAS	0.05 *μ*M	—	SRMs (DOLT-4 and DORM-2) were analyzed. The RSD values in the determination of Hg(II) was less than 10%	Sea food samples	Majority of cations did not interfere in the determination of Hg(II) at pH 6.25 experimental condition	[54]

Total Hg	Solid sampling	—	HR-AAS	2.0 × 10^−5 ^*μ*g (sediment), 9.6 × 10^−5 ^*μ*g (marine biota)	2.0 × 10^−5^–0.004 *μ*g (sediment), 2.0 × 10^−5^–0.025 *μ*g (marine biota)	CRMs (PACS-2, IAEA-405, SRM 2703, BCR-464, IAEA-436, DORM-2, MA-ROPME-2/TM) were analyzed	Sediment and marine biota samples	—	[55]

Total Hg	Thermal desorption	—	AAS	0.0006 *μ*g g^−1^	—	CRMs (CRM-1515, MESS-3 and TORT-2) were analyzed with recoveries 96.0–104.8%	Fish and sea food samples	—	[60]

Total Hg	ISO guide 34	—	CV-ICP-MS	8.0 × 10^−5 ^*μ*g/g	1.9–50 × 10^−5 ^*μ*g/g	CRM (BCR-579) was analyzed	Sea water	By using cold vapor generation spectral interferences were avoided	[61]

Total Hg	Acid digestion	Nitric and perchloric acid	AAS	0.0004749 *μ*g/g	0.0002–0.01 *μ*M	The mean recovery of Hg was 78.65%. RSD values for interday precision of Hg was 7.17%	Cream cosmetics	Along with Hg, zinc was also determined	[109]

Total Hg	Extraction	NaBH_4_	CV-AAS	0.003 *μ*M	0.05–0.5 *μ*M	The recoveries of Hg in oil samples were in the range of 80–103%	Oil samples	—	[104]

Total Hg	Thermal desorption	—	TDA-AAS	0.025 *μ*g/g (LOQ)	—	Mean recovery of Hg in real samples was 94.2% and SD was 3.5%	Sea food	—	[105]

Total Hg	Method EPA 7473	Teflon	CV-AFS	0.0006 *μ*g g^−1^	0.002–0.08 *μ*g·g^−1^	CRM (BCR-279) was analyzed and RSD in the determination of Hg in seaweeds is less than 10%	Seaweeds	—	[62]

Hg(II)	Immobilization	Dithizone	FAAS	2.0 × 10^−9^ M	1.1 × 10^−8^–2.0 × 10^−6 ^M	The coefficients of variation for Hg(II) was found to be 2.7%	Industrial wastewater, spiked tap water, and natural water	Except Cu^2+^, the other ions (Mn^2+^, Ni^2+^, Pb^2+^, Co^2+^, Cd^2+^, Fe^2+^, Fe^3+^, and Al^3+^) did not interfere up to 50-fold excess	[114]

MeHg	Online preconcentration	—	HPLC-CV-AFS	40000 *μ*M	5–2500 *μ*M	CRMs (NIES CRM no. 13 and IAEA-085) were analyzed. Recoveries of MeHg from real samples were in the range of 91.4–101.8%	Sewage, river, and seawater samples	—	[47]

Hg(II)	Solid phase extraction	Ion imprinted polymeric nanomaterials	CV-AAS	0.18 *μ*M	—	The RSD values for eight replicates was 4.2%	Water and human hair samples	In presence of large amounts of Cu^2+^, Ni^2+^, Cd^2+^, Zn^2+^, Mn^2+^, Pb^2+^, Fe^3+^, and Cr^3+^ ion Hg(II) was effectively determined	[115]

Hg(II), MeHg	Rapid extraction	—	HPLC-ICPMS	0.0002 (Hg2+), 0.0001 (MeHg) *μ*g g^−1^	—	CRMs (TORT-2 and DORM-2) were analyzed	Fish samples	—	[63]

Speciation	Liquid-liquid microextraction	Ionic liquid vortex-assisted	HPLC-CV-AFS	3.4–6.1 × 10^−6^ *μ*g/g	0.0001–0.07 *μ*g/g	The RSD values were less than 6.4%	Sediment samples	—	[92]

Total Hg	Solid sampling	—	TDA-AAS	0.001 *μ*g g^−1^	0.025–0.2 *μ*g g^−1^	Recoveries of Hg from real samples were in the range of 89–99%	Fish and shrimp samples	—	[106]

Hg speciation	Cloud point extraction	Polyethylene glycol	Spectrophotometer	0.045 *μ*M	0.05–0.5 *μ*M	The RSD values of the method were below 2.6%	River water and river sediment	No interference of Cd2+, Bi3+, and Pb2+ was observed in the determination of Hg(II)	[107]

Hg(II)	Colorimetric	1,5-diphenylthiocarbazone	Flow injection spectrophotometer	0.15 *μ*M	0.25–7.5 *μ*M	The results were compared with the data obtained with ICP-MS	Cosmetics and Thai traditional medicines	20-fold Fe^2+^, Zn^2+^, and Cu^2+^, 40-fold Pb^2+^, 50-fold Al^3+^, Fe^3+^, and Mn^2+^ did not interfere in the determination of Hg(II)	[94]

Hg(II)	Colorimetric, fluorescence	Calixpyrrole hydrazide	Spectrofluorometer	1 nM	1 nM–1 *μ*M	—	Groundwater and industrial effluent water	No interference of Pb(II), Cd(II), Mn(II), Fe(III), Ni(II), Zn(II), Hg(II), Co(II), and Cu(II) was observed	[70]

Hg(II)	Fluorescence probe	Chitosan hydrogel	Fluorescence spectrophotometer	0.9 nM	5.0–50 nM	—	Water samples	Cations such as Fe^3+^, Co^2+^, Pb^2+^, Cu^2+^, Cd^2+^, Ni^2+^, and Zn^2+^ did not interfere in the determination of Hg(II)	[71]

Hg(II)	Fluorescence sensor	CdTe quantum dots	Fluorescence spectrophotometer	4.0 nM	6.0–450 nM	RSD values were less than 4.15%	Lake water samples	Interference of 10-fold Pb^2+^, Cu^2+^, and Ag^+^ was less than 7%	[116]

Hg(II)	Fluorescent chemosensor	DA	Fluorescence spectrophotometer	0.0028 *μ*l/L	—	Theoretical and experimental results were in good agreement with each other	—	Simultaneous determination of Ag+ and Cu2+ was reported	[98]

Hg(II)	Time-gated fluorescent sensing	Thymine	Spectrofluorometer	0.11 nM	0.20–10 nM	Recoveries of Hg(II) in environmental water samples were in the range of 93.75–102.5%	Drinking water samples	No interference of Ag+, Co^2+^, Ni^2+^, Ca^2+^, Cd^2+^, Al^3+^, Fe^3+^, Au^3+^, Cr^2+^, Mn^2+^, Pb^2+^, Cu^2+^, Mg^2+^, Zn^2+^, and Ba^2+^ was reported	[117]

Hg(II)	Colorimetric	Gold nanoparticles	Spectrophotometer	0.5 nM	0.5–300 nM	CRM (GBW (E) 080392) was analyzed and the recoveries were found in the range of 88.9–106%	Tap water and lake water	Cr^3+^, Mn^2+^, Co^2+^, Ni^2+^, Cu^2+^, Fe^3+^, Zn^2+^, Cd^2+^, and Pb^2+^ ions did not interfere in the determination of Hg^2+^	[64]

Hg(II)	Fluorescence	Schiff base	Fluorescence spectrophotometer	2.82 × 10^−6^ M	—	Effectiveness of the method was proved by confocal fluorescence microscope	Living cells	—	[95]

Hg(II)	Colorimetric	Silver nanoparticles	Spectrophotometer	1.18 × 10^−9^ M	10–50 nM	A good linear correlation (*R*^2^ = 0.9799) was obtained for different concentrations of Hg(II) and absorbance ratio	Lake, seawater, and groundwater	Fe^2+^, Fe^3+^, Cr^6+^, Pb^2+^, Mn^2+^, Al^3+^, Ni^2+^, Cr^3+^, Cd^2+^, Mg^2+^, and Zn^2+^ did not interfere up to 1000 times of detection limit of Hg(II)	[118]

Hg(II)	Chemosensor	Dimeric binol-based chemosensor	Spectrofluorometer	4.4 × 10^−7 ^M	—	—	Wastewater samples	100 equivalents of Na^+^, K^+^, Mg^2+^, Ce^3+^, Ca^2+^, Ba^2+^, Cd^2+^, Mn^2+^, Co^2+^, Ni^2+^, Cu^2+^, Cr^3+^, Zn^2+^, Pb^2+^, Fe^2+^, Fe^3+^, Al^3+^, and Ag^+^	[72]

Hg(II)	Colorimetric	Bovine serum albumin	Spectrophotometer	7.2 nM	0–120 nM	Results were compared with ICP-MS	Drinking water samples	No interference of Na^+^, Mg^2+^, Ca^2+^, Cd^2+^, Mn^2+^, Co^2+^, Ni^2+^, Cu^2+^, Zn^2+^, Pb^2+^, Fe^3+^, and Au^3+^ was observed	[119]

Hg(II)	Fluorescence	Gold nanocluster	Spectrofluorometer	30 nM	—	Recoveries of Hg(II) in spiked samples were in the range of 97.7–99.3%	Lake water samples	No interference of Na^+^, Mg^2+^, Ca^2+^, Ni^2+^, Cu^2+^, Zn^2+^, Ce^3+^, Pt^4+^, and Al^3+^ was observed	[120]

Hg(II)	Colorimetric	Rhodamine B	Spectrofluorometer	1.71 × 10^−6 ^M	—	—	Spiked tap water samples	Interference of several ions was negligible in the determination of Hg(II)	[73]

Hg(II)	Fluorescence	—	Fluorescence spectrometer	9.56 × 10^−9^ M	—	—	—	Hg(II) can be detectable in presence of Fe^3+^, Cu^2+^, Co^2+^, Ni^2+^, Cd^2+^, Pb^2+^, Zn^2+^, and Cr^3+^	[74]

Hg(II)	Adsorption	Rhodamine	Fluorescence spectrophotometer	3.42 × 10^−6^ M	0–6.0 *μ*M	—	Drinking and lake water	No interference of Cd^2+^, Co^2+^, Cu^2+^, Fe^3+^, Mn^2+^, Ni^2+^, Pb^2+^, and Zn^2+^ at 581 nm	[75]

Hg(II)	Fluorimetric	Coumarinyldithiolane	Fluorescence spectrophotometer	—	0.06–1.5 *μ*M	—	Aqueous solutions	No influence of Al^3+^, Zn^2+^, Co^2+^, Ni^2+^, Cu^2+^, Cd^2+^, Cr^3+^, and Pb^2+^ on the determination of Hg(II) in presence of probe	[76]

Hg(II)	Fluorescence sensors	Peanut shell	Fluorescence spectrometer	8.5 × 10^−9 ^M	0–19 × 10^−8 ^M	—	Lake water	The method was selective for Hg(II)	[77]

Hg(II)	Colorimetric	L-Arginine	Spectrophotometer	5 nM	1–20 and 20–600 *μ*M	—	Food samples	No interference from Cd^2+^, Co^2+^, Cu^2+^, Ni^2+^, and Pb^2+^ was observed	[78]

LLME: Liquid-liquid microextraction; CRM: certified reference material; CVG: chemical vapor generation; THB: tetrahydroborate; RSD: relative standard deviation; LOQ: limit of quantification; MIP-OES: microwave-induced plasma optical emission spectrometer; PPT: poly(1,4-bis-(8-(4-phenylthiazole-2-thiol)-octyloxy)-benzene); LSPR: localized surface plasmon resonance; DA: dimethylaminocinnamaldehyde-aminothiourea. *Analytical Instruments*. CV-AAS: Cloud Vapor Atomic Absorption Spectrometer; GF-AAS: Graphite Furnace AAS; ICP-OES: Inductively Coupled Plasma Optical Emission Spectrometer; ICP-MS: ICP-Mass Spectrometer; ICP-AES: ICP-Atomic Emission Spectrometer; HPLC: High Performance Liquid Chromatography; AFS: Atomic Fluorescence Spectrometer.

**Table 2:** Analytical parameters of reviewed research papers involving speciation and determination of mercury by electrochemical instruments.

Analyte	Method	Supporting media	Analytical instrument	LOD	Linearity range	QA/QC studies	Analyzed samples	Interference study	Ref
Hg(II)	Biosensor	Y-shaped DNA	Square wave voltammeter	0.094 nM	1 nM–5 *μ*M	Selectivity, sensitivity, and repeatability were studied	River water samples	Interferences of Cu^2+^, Al^3+^, Co^2+^, Fe3^+^, Zn^2+^, Ni^2+^, Cd^2+^, Ba^2+^, Cr^3+^, Mg^2+^, and Pb^2+^ were reported	[121]

Hg(II)	Preconcentration	N-Octylpyridinium	Stripping voltammeter	0.0015 *μ*M	0–0.5 *μ*M	The RSD of the method was 10%	Tap, pond, and wastewaters	No significant interference of 100 *μ*g L^−1^ of Cu^2+^, Pb^2+^, Cd^2+^, and Zn^2+^ was observed in the determination of Hg^2+^	[122]

Hg(II)	Electrochemical	Screen printed carbon electrode	Anodic stripping voltammeter	0.005 *μ*M	0.005–0.5 *μ*M	Accuracy of the method was evaluated with ICP/MS	Groundwater	Interference of Cu^2+^, Co^2+^, Fe^2+^, Zn^2+^, Ni^2+^, Cd^2+^, Mn^2+^, Mg^2+^, and Pb^2+^ was negligible in the determination of Hg^2+^	[123]

Hg(II)	Electrochemical sensor	1-(2, 4-Dinitrophenyl)-dodecanoyl thiourea	Cyclic, square wave and differential pulse voltammeter	0.0032 *μ*M	Up to 0.01 *μ*M	The RSD of the method was 3.5%	Drinking and tap water samples	5-fold Cu^2+^, Cd^2+^, Pb^2+^, and Zn^2+^ did not interfere in the determination of Hg(II)	[124]

Hg(II)	Electrochemical	N-PC-Au	Anodic stripping voltammeter	0.35 nM	0.001–1 *μ*M	—	Drinking water	The electrode was not affected by the presence of Zn^2+^, Pb^2+^, Cu^2+^, and Cd^2+^ ions in the determination of Hg(II)	[79]

Hg(II)	Electrochemical sensor	Modified gold nanoparticles	Cyclic voltammeter	7.5 *μ*M	5.0–50 *μ*M	—	Spiked water samples	The method is selective towards the presence of Zn^2+^, Cd^2+^, Pb^2+^, Cu^2+^, Ni^2+^, and Co^2+^ ions	[80]

Hg(II)	Electrochemical	N-doped graphene electrode	Differential pulse voltammeter	0.05 *μ*M	0.2–9 *μ*M	The RSD of Hg determination with six repetitions was 2.1%		Simultaneously Cd^2+^, Cu^2+^, and Pb^2+^ were determined along with Hg^2+^	[99]

Hg(II)	Electrochemical sensor	Screen printed carbon electrode	Differential pulse anodic stripping voltammeter	0.0001 *μ*M	0.0002–0.01 *μ*M	Recovery of Hg(II) was found as 106%	Real water samples	High tolerance limits were observed for Fe^3+^, Zn^2+^, and Cd^2+^ but lower tolerance limits for Pb^2+^ and Cu^2+^ were found	[125]

Hg(II)	Electrochemical sensor	DNA probe	Cyclic and square wave voltammeter	5.6 nM	10–100 nM	—	—	10-fold Pb^2+^, Mn^2+^, Zn^2+^, Ni^2+^, Cu^2+^, Fe^2+^, Ba^2+^, and Cd^2+^ did not interfere in the determination of Hg(II)	[81]

Hg(II)	Electrochemical	Carbon ionic liquid paste electrode	Anodic stripping voltammeter	0.1 nM	0.5–10 nM and 0.08–2 *μ*M	—	Wastewater samples	Over 30-fold Zn2+, Cr3+, and Pb2+ and over 45-fold Cd2+, Cu2+, Ni2+, and Mn2+ interfered in the determination of Hg(II)	[82]

Hg(II)	Electrochemical	Carbon paste sensor	Potentiometer	1.95 × 10^−9 ^M	4.00 × 10^−9^–1.30 × 10^−3 ^M	Reproducibility of the method was reported	Water samples	Selective coefficients of various cations for Hg(II) selective sensors were reported	[126]

Hg(II)	Biosensor	Thymine	Differential pulse and cyclic voltammeter	0.08 nM	0.5–5000 nM	Recoveries of Hg(II) in real samples were in the range of 96.4–103%	Water and human serum	Selective in presence of Al^3+^, Ba^2+^, Cd^2+^, Co^2+^, Cr^3+^, Fe^3+^, Mn^2+^, Pb^2+^, and Zn^2+^	[127]

Hg(II)	Biosensor	Cyclic dithiothreitol	Cyclic voltammeter	28 pM	0.1 nM–5 *μ*M	Recoveries of Hg(II) in water samples were in the range of 98.8–104%	River water samples	Excellent selectivity for Hg(II) detection was observed in presence of Cd^2+^, Pd^2+^, and Co^2+^	[128]

Hg(II)	Biosensor	Methylene blue	Cyclic voltammeter	8.7 × 10^−11 ^M	1.0 × 10^−10^–5.0 × 10^−7^ M	The RSD of the sensor was 5.25% for 10 replicates indicating the good reproducibility	Tap and river water samples	Cd^2+^, Ba^2+^, Pb^2+^, Ni^2+^, Cu^2+^, Zn^2+^, Mn^2+^, Ca^2+^, Co^2+^, Mg^2+^, and Ag^+^ did not interfere up to 250 nM in presence of 50 nM of Hg(II)	[129]

Hg(II)	Electrochemical	PVC membrane sensor	Potentiometer	3.2 × 10^−9^ M	1.0 × 10^−8^–5.0 × 10^−3 ^M	RSD values for synthetic samples measurements were less than 3.10%	Wastewater samples	The selectivity coefficients for various ions were in the range of 1.0 × 10^−4^–4.5 × 10^−4^ M	[130]

Hg(II)	Electrochemical	Copper film electrode	Anodic stripping voltammeter	0.0005 *μ*M	0.05–0.5 *μ*M	The RSD value for 12 replicates of Hg determination was 4.5%	—	Simultaneously mercury and lead are determined	[100]

Hg(II)	Electrochemical	Carbon nanotubes	Anodic stripping voltammeter	0.025 *μ*M	0.1–100 *μ*M	The RSD value for six replicates was 1.93%	River and industrial wastewater	Up to 200-fold Pb^2+^, Cu^2+^, Cd^2+^, Zn^2+^, Ni^2+^, and Mn^2+^ did not interfere in the determination of Hg(II)	[131]

Hg(II)	Electrochemical sensor	Mesoporous carbon nanofibre	Anodic stripping voltammeter	0.3 nM	5–500 nM	The RSD values in the determination of Hg(II) in real samples were less than 2.3%	Yellow river, China	The proposed electrode avoids the interferences of Cd^2+^, Pb^2+^, and Cu^2+^	[132]

Hg(II)	Potentiometric sensor	MWCNTs	Potentiometer	3.1 × 10^−9 ^M	4.0 × 10^−9^–2.2 × 10^−3^ M	The recoveries of Hg(II) were in the range of 99–102%	Aqueous samples	The proposed method was highly selective towards the determination of Hg(II) in presence of some other interfering ions in aqueous samples	[133]

Hg(II)	Electrochemical	Rotating silver electrode	Square wave voltammeter	4.61 × 10^−8 ^M	1.0 × 10^−7^–8.0 × 10^−4^ M	The RSD for seven replicates was 2.19%	Milk and breast milk	No interferences of copper, cobalt, iron, and zinc were observed	[134]

Hg(II)	Electrochemical	Graphene modified with silver	Differential pulse voltammeter	3.38 × 10^−8 ^M	5.0 × 10^−8^–1.0 × 10^−4 ^M	The RSD for eight replicates was 2.25%	Leachate samples	Even 200 times excess of Al^3+^, Cd^2+^, Co^2+^, Ni^2+^, Pb^2+^, Fe^2+^, Fe^3+^, and Zn^2+^ did not interfere	[135]

Hg(II)	Electrochemical	Graphene oxide	Cyclic voltammeter	0.035 nM	0.1–100 nM	The RSD value in the reproducible test was 4.5%	River water samples	Even 10 times higher concentrations of Co^2+^, Mn^2+^, Pb^2+^, and Fe^3+^ did not interfere in the determination of Hg(II)	[136]

Hg(II)	Electrochemical	Gold nanoparticles	Differential pulse anodic stripping voltammeter	0.0001 *μ*M	0.0005–0.05 *μ*M	Recoveries of Hg(II) in real samples were in the range of 87–102%	Tap and lake waters, milk, and soils	1000-fold Zn^2+^, Cd^2+^, Pb^2+^, Mn^2+^, Co^2+^, and Cu^2+^ did not interfere in the determination of Hg(II)	[137]

Hg(II)	Electrochemical	Gold nanoparticles	Stripping voltammeter	1 *μ*M	—		Water samples	—	[83]

Hg(II)	Electrochemical	Graphene-Au modified electrode	Square wave voltammeter	0.001 aM	1.0 aM–100 nM	The RSD values for triplicate measurements was less than 4.46%	Spiked tap and river waters and landfill leachate samples	Even 500 nM of Cd^2+^, Co^2+^, Cr^2+^, Cu^2+^, Mn^2+^, Ni^2+^, Pb^2+^, Zn^2+^, Al^3+^, and Fe^3+^ did not interfere in the determination of 10 nM of Hg(II)	[48]

Hg(II)	Electrochemical	Graphene/CeO_2_	Differential pulse anodic stripping voltammeter	2.187 × 10^−11^ M	0.002–0.12 *μ*M	—	Wastewaters	Simultaneously Cd^2+^, Pb^2+^, Cu^2+^, and Hg^2+^ were determined	[84]

Hg(II)	Electrochemical	Graphene quantum dots	Anodic stripping voltammeter	0.02 nM	0.02–1.5 nM	Recoveries from spiked samples were in the range of 96.6–101%	Spiked samples	Cu^2+^ was also determined along with Hg(II)	[138]

Total Hg	Liquid-liquid microextraction	Screen printed carbon electrodes	Square wave anodic stripping voltammeter	0.00005 *μ*M	0.0025–0.05 *μ*M	The recoveries in the determination of mercury in real samples were in the range of 95–108%	Tap, river, and bottled and industrial wastewaters	—	[139]

Total Hg	Electrochemical sensing	Zinc oxide quantum dots	Linear sweep voltammeter	0.005 *μ*l/L	0.005–0.05 *μ*l/L	—	River and groundwater	Except Cd^2+^, the other ions, such as Zn^2+^, Pb^2+^, and As^3+^ did not interfere	[85]

Total Hg	Electrochemical	Gold nanoparticles	Quartz crystal microbalance	0.15 nM	3–300 nM	The results were compared with CV-AAS technique. The RSD was found to be less than 7%	Water and sediment samples	Interference of Cu^2+^, Cr^3+^, Pb^2+^, and Cd^2+^ was reported	[140]

Hg(0)	Electrochemical	Gold-based microsensor	Quartz crystal microbalance	—	—	The results were accurate and within 8% of the concentrations reported by EPA certified samples	Industrial flue gas	—	[141]

Hg(0)	Electromechanical	—	Quartz crystal microbalance	2.42 × 10^−8^ *μ*M	—	Selectivity of the instruments for mercury was 84%	—	—	[142]

Hg(0)	Electrochemical	Silver/gold core/shell nanowire monolayer	Quartz crystal microbalance	0.039 *μ*M	—	Repeatability of the results was always greater than 87%	Industrial gas effluents	—	[143]

N-PC-Au: nitrogen-doped porous carbon-gold nanocomposite; MWCNTs: multiwalled carbon nanotubes.

**Table 3:** Analytical parameters of reviewed research papers involving speciation and determination of mercury by miscellaneous techniques.

Analyte	Method	Supporting media	Analytical instrument	LOD	Linearity range	QA/QC studies	Analyzed samples	Interference study	Ref
Hg(II)	Colorimetric	Gold nanoparticles	Dark-field microscope	1.4 pM	—	Recoveries were 98.3 and 110.0% for river and industrial wastewater, respectively	River and industrial wastewater	25 nM concentrations of Pb^2+^, Ni^2+^, Fe^2+^, Cd^2+^, Zn^2+^, Co^2+^, and Mn^2+^ did not interfere in the determination of Hg (II)	[49]

Hg(II)	Electrochemiluminescence	Gold nanoparticles	Potentiostat PG340	5.1 pM	—	Results were compared with AFS measurements	Tap and lake waters	The method was selective for Hg determination in presence of Cd^2+^, Co^2+^, Cu^2+^, Fe^2+^, Mg^2+^, Mn^2+^, Pb^2+^, Al^3+^, and Fe^3+^	[50]

Hg(0)	Thermal desorption	—	Direct milestone analyzer	—	—	Accuracy was verified with testing the SRM (NIST-2711) and CRM (GBW-GBW 08301 RCV 8221)	Soil samples	—	[56]

Hg(II)	Electrochemiluminescence	*γ*-Polyglutamic acid-grapheme-luminol	Chemiluminescence analyzer	1.0 × 10^−6 ^*μ*l/L	2.0 × 10^−6^–0.02 *μ*l/L	The RSD values for reproducibility of biosensor were 6.2%; the results were compared with ICP-MS	River water samples	No interference of Pb^2+^, Zn^2+^, Cu^2+^, Mg^2+^, and Cd^2+^ was observed	[144]

Hg speciation	Liquid-liquid-liquid microextraction	18-crown-6	Electrophoresis	0.005–0.03 (Hg^2+^), 0.004–0.027 (Me Hg), 0.001–0.0075 (PhHg) *μ*M	0.01–1 *μ*M	The RSD values of the reproducibility tests were less than 13.0%	Hair and water samples	—	[90]

Hg(0)	UV-light generation	Multimode diode lasers	Photomultiplier modules	0.12 *μ*M	0–60 *μ*M	The coefficient of linear regression was obtained as *R*^2^ = 0.998	—	—	[101]

Total Hg	—	—	Direct mercury analyzer	—	—	—	Fish samples	Vanadium also determined along with mercury	[86]

MeHg	ISO-17025	—	Advanced mercury analyzer	9.0 × 10^−6 ^*μ*g	9.0 × 10^−6^–0.003 *μ*g	CRMs (IAEA-436, DOLT-2, TORT-2, IAEA-452) were analyzed; the RSD values were in the range of 1.7–4.5%	Marine biota samples	—	[65]

Total Hg	—	—	Direct mercury analyzer	0.0027 *μ*g/g	0.002–0.15 *μ*g	Recoveries of Hg were in the range of 98.9–106.1%; CRM (DORM-3) was analyzed	Animal tissues	—	[66]

Total Hg	—	—	Direct mercury analyzer (DMA-80)		0–50 ng	SRM (NIST-1633b) and Rice fluor-NIES-10 (Japan) were analyzed	Human hair and nails	—	[57]

Total Hg	Platinum trap	—	Combustion mercury analyzer (MA 3000)	0.00027 *μ*g/g	—	CRMs (INCT-PVTL-6) and STRP-IS3 were analyzed	Tobacco samples	—	[67]

Total Hg	Colorimetric	Lysine	Anisotropic gold nanoparticles	27 pM	0.01–1.0 nM	—	Deionized and tap waters	No interference of As^3+^, Cr^3+^, Cd^2+^, Pb^2+^, Ni^2+^, Zn^2+^, and Ba^2+^ was observed	[51]

Total Hg	US EPA method 7473		Direct mercury analyzer	—	—	Method was compared with TD-AAS results	Sediments	—	[93]

Hg(II)	Electrochemiluminescent	Graphene coupled quantum dots	MPI-A multifunctional electrochemical analytical system	0.0003 *μ*M	0.2–5 *μ*M	The RSD values in the determination of Hg(II) real samples were in the range of 2.4–7.5%	Spiked and real water samples	No interference of Cu^2+^, Pb^2+^, Ni^2+^, and Cd^2+^ was observed	[145]

Hg(II)	Photoelectrochemical	CdS quantum dots	Atomic force microscope	6.0 × 10^−10 ^M	3.0 × 10^−9^–1.0 × 10^−7 ^M	—	—	100-fold Cr^3+^, Fe^3+^, Pb^2+^, Cd^2+^, Cu^2+^, Mn^2+^, Zn^2+^, Al^3+^, and Co^3+^ did not interfere	[87]
